# *Pasteurella multocida* activates Rassf1-Hippo-Yap pathway to induce pulmonary epithelial apoptosis

**DOI:** 10.1186/s13567-024-01285-y

**Published:** 2024-03-16

**Authors:** Guangfu Zhao, Yunhan Tang, Xiongli Liu, Pan Li, Tianci Zhang, Nengzhang Li, Fang He, Yuanyi Peng

**Affiliations:** 1 College of Veterinary Medicine, Southwest University, Chongqing, China; 2 Department of Environment and Safety Engineering, Taiyuan Institute of Technology, Taiyuan, China; 3 Department of Endocrinology and Metabolism, Center for Diabetes and Metabolism Research, West China Hospital, Sichuan University, Chengdu, China

**Keywords:** *Pasteurella multocida*, pulmonary epithelium, Rassf1-Hippo-Yap pathway, apoptosis, pathogenesis

## Abstract

*Pasteurella multocida* is an opportunistic zoonotic pathogen that primarily causes fatal respiratory diseases, such as pneumonia and respiratory syndromes. However, the precise mechanistic understanding of how *P. multocida* disrupts the epithelial barrier in mammalian lung remains largely unknown. In this study, using unbiased RNA-seq analysis, we found that the evolutionarily conserved Hippo-Yap pathway was dysregulated after *P. multocida* infection. Given the complexity of *P. multocida* infection associated with lung injury and systemic inflammatory processes, we employed a combination of cell culture models, mouse models, and rabbit models to investigate the dynamics of the Hippo-Yap pathway during *P. multocida* infection. Our findings reveal that *P. multocida* infection activates the Hippo-Yap pathway both in vitro and in vivo, by upregulating the upstream factors p-Mst1/2, p-Lats1, and p-Yap, and downregulating the downstream effectors *Birc5*, *Cyr61*, and *Slug*. Conversely, pharmacological inhibition of the Hippo pathway by XMU-MP-1 significantly rescued pulmonary epithelial cell apoptosis in vitro and reduced lung injury, systemic inflammation, and mouse mortality in vivo. Mechanistic studies revealed that *P. multocida* induced up-regulation of Rassf1 expression, and Rassf1 enhanced Hippo-Yap pathway through phosphorylation. Accordingly, in vitro knockdown of Rassf1 significantly enhanced Yap activity and expression of Yap downstream factors and reduced apoptosis during *P. multocida* infection. *P. multocida*-infected rabbit samples also showed overexpression of Rassf1, p-Lats1, and p-Yap, suggesting that *P. multocida* activates the Rassf1-Hippo-Yap pathway. These results elucidate the pathogenic role of the Rassf1-Hippo-Yap pathway in *P. multocida* infection and suggest that this pathway has the potential to be a drug target for the treatment of pasteurellosis.

**Supplementary Information:**

The online version contains supplementary material available at 10.1186/s13567-024-01285-y.

## Introduction

*Pasteurella multocida* (Pm) is gram-negative pathogen that can be fatal to both wild and domestic animals, including rabbits, cattle, chickens and pigs [1, 2]. There have even been cases of human infection [3, 4]. Pm can be classified into 5 serotypes, based on their capsule characteristics: A, B, D, E and F [5]. Among these, Pm serogroup A (PmA), which is usually found in domestic animals, especially in cattle, rabbits and chickens, is known to cause fatal pneumonia, resulting in significant economic losses in animal husbandry worldwide [6–10]. Currently, antibiotics are the main treatment option for pasteurellosis due to the lack of effective vaccines and specific drugs. Additionally, there has been an increase in human pasteurellosis cases over the past 20 years, making Pm a potential public health risk factor [11]. Therefore, there is an urgent need for further research to investigate the pathogenesis of Pm.

Clinically, Pm (especially PmA)-induced pneumonia is characterized by increased bacterial load in the lungs, extensive alveolar epithelial damage, and massive infiltration of alveolar leukocytes [12]. In our previous studies, we reproduced Pm-induced pneumonia based on a mouse model and focused on the role of immune cells such as macrophages and T cells in the pathogenesis of Pm [6, 13–15]. In fact, lung epithelial cells play a crucial role in defense against infection, tissue repair and regeneration, and inflammatory regulation. However, little attention has been paid to how Pm induces lung epithelial damages. Some researchers have found that alveolar epithelial cell death (including apoptosis) was caused by Pm, but the underlying mechanism is largely unknown [16, 17]. As with other pathogens, the pathogenic mechanism of Pm must be a complex process involving crosstalk with various cell signaling pathways. To understand the fundamentals of the Pm-mediated cellular injury process, we reviewed previously published RNA sequence data [13], and then speculated the cellular Hippo pathway, a highly conserved pathway involved in the regulation of tissue growth, tissue repair and regeneration, inflammation, and anti-apoptosis, might be involved in the pathogenic process of Pm [18, 19]. Dysregulation of this signaling pathway has also been observed in other bacterial or viral pneumonia pathogens such as *Streptococcus pneumoniae* and COVID-19 [20, 21].

The core components of the Hippo pathway consist of upstream kinase cascades and downstream effectors. The kinase cascades of Hippo pathway in mammal mainly include STE20-like kinase 1/2 (MST1/2) and large tumor suppressor 1/2 (LATS1/2) [22, 23]. Once activated by stimuli, LATS1/2 are phosphorylated and activated by the upstream factors MST1/2. Phosphorylated LATS1/2, then phosphorylate downstream effector Yes-associated protein (YAP), which is well-known as a transcription factor and oncogene [24, 25]. Because it is phosphorylated, YAP is retained in the cytoplasm, and subsequently ubiquitinated and degraded by the proteasome. On the opposite, Hippo pathway inactivation allows cytoplasm unphosphorylated YAP together with transcriptional coactivator with PDZ-binding motif (TAZ) being translocated in the nucleus, where they interact with TEAD1-4 and induce expression of target genes involved in tissue repair, cell proliferation and cell survival, such as cysteine-rich angiogenic inducer 61 (Cyr61) and snail family transcriptional repressor 2 (Snai2 or Slug) [26, 27]. The Hippo pathway has been shown to be closely related to respiratory diseases due to its important role in regulating cell proliferation, survival, organ size, tissue development, repair and regeneration [28]. Studies have also found that the Hippo pathway regulates bacterial pneumonia, in which YAP exerts anti-infective effects by regulating cell death, tissue regeneration and inflammatory responses [29, 30]. Therefore, we hypothesize that the Hippo pathway may play a crucial role in the pathogenesis of Pm.

Here, we demonstrate the involvement of Hippo-Yap pathway activation in the pathogenic process of Pm based on a mouse model using unbiased RNA-seq technology. In addition, we identify the mechanism by which Pm induces Hippo-Yap pathway activation and demonstrate that the Hippo-Yap pathway could be a potential target for pasteurellosis and other respiratory bacterial diseases.

## Materials and methods

### Reagents, plasmids, primers, and antibodies

Detailed information on the reagents, antibodies, plasmids and primers used in this study are presented in Additional file 5.

### Bacterial strains and cultivation condition

The *Pasteurella multocida* serogroup A CQ2 (GenBank accession number: No. CP033599) was isolated from cattle in Chongqing, China, and cultured on Martin’s broth agar plus 5% horse serum at 37 ℃. Mouse immortalized lung epithelial cell line TC-1 and Lewis lung carcinoma cell line LLC were bought from ATCC (American Tissue Culture Collection, Beijing) and cultivated in Dulbecco’s modified Eagle medium high glucose (DMEM-H, Hyclonal) supplemented with 10% fetal bovine serum at 37 ℃ under 5% CO_2_.

### Western blotting

Cells in culture wells were lysed with RIPA buffer containing protease inhibitor cocktail. For tissues, a homogenization step is required. Proteins in the supernatant were obtained by centrifugation at 4 °C. The protein concentration in the supernatant was quantified by the BCA method (Thermo Fisher, USA) and then boiled with a reducing agent (Dithiothreitol, DTT, Solarbio, China). Protein samples were separated by SDS-PAGE, transferred to PVDF membranes, blocked with 5% skimmed milk and incubated with the corresponding primary antibodies. Finally, PVDF membranes were incubated with the corresponding HRP-conjugated secondary antibodies (Boster Bio, BA1054, BA1050, and BA1060) and then visualized with an ECL kit (Bio-Rad, USA).

### Annexin V/PI apoptosis assay

Harvested cells were stained with Annexin V-FITC and PI according to the instructions of the manufacturer of the Annexin V-FITC/PI Apoptosis Kit (Elabscience, E-CK-A211). Finally, apoptotic cells were quantified by flow cytometry.

### Transwell assay

The assay was performed using 8.0 µm pore size Transwell inserts (Corning, 3422). Briefly, antibiotic-free medium with or without drug was added to both the upper and lower chambers and 1 × 10^5^ cells were seeded in the upper chamber for 24 h, then 1 MOI PmCQ2 and the appropriate drug was added to the upper chamber and the cells were incubated. After 8 h of incubation, the bottom and top media were collected and diluted to appropriate dilution and the number of translocated bacteria was measured by plate counting method. Translocation rate = number of transit bacteria/ (number of upper bacteria + number of transit bacteria) × 100%.

### Animal experiment

Male ICR mice (CD-1 mice) at age of 6–8 weeks, weighting 25–30 g, and male New Zealand rabbits at age of 2-month-old, weighting 2000 g, were purchased from Byrness Weil biotech Ltd (Chongqing, China). Mice and rabbits were housed in independent airy cages at 25 ℃ with free access to water and food. All experimental animal protocols followed The Guide for the Care and Use of Laboratory Animals published by the National Institutes of Health (NIH). The animal protocol used in this study has been granted permission by the Ethics Committee of Southwest University, No. LAC2023-2-0232. Animals were randomly assigned to each experimental group.

Pm infection mouse model was generated by intranasal infection with 10^4^ CFU log-phase growth PmCQ2. For intranasal infection, 1.5% pentobarbital sodium were previously used to anesthetize mice. Pm infection rabbit model was generated by intranasal infection with 10^9^ CFU log-phase growth PmCQ2. All animal experiments except survival curves experiments were conducted at least 3 times. For the survival curves experiments, the PmCQ2 infection experiments were conducted 3 times, and representative survival curves were shown.

### Quantitative real-time PCR

AFTSpin Tissue/Cell Fast RNA Extraction Kit for Animals (ABclonal, RK30120) was used to harvest the total RNA. The harvested RNA was then reverse transcribed into a cDNA pool with ABScript III RT Master Mix for qPCR with gDNA Remover (ABclonal, RK20429). Real-time PCR was performed by Bio-Rad CFX96. The relative mRNA expression was normalized to the expression of beta-actin mRNA.

### RNA-sequencing

The RNA libraries were sequenced by OE Biotech, Inc., Shanghai, China. Briefly, three wild type mouse lungs and three mouse lungs infected with 10^4^ CFU PmCQ2 for 24 h were homogenized in liquid nitrogen, and then total RNA was extracted using TRIzol reagent (Invitrogen). rRNA was removed using the Ribo-Zero rRNA Removal Kit (Illumina, MRZH116). RNA integrity was assessed using the Agilent 2100 Bioanalyzer. Each specimen made up a total of 1 μg of the cDNA library. Then the libraries were constructed using VAHTS Universal V6 RNA-seq Library Prep Kit according to the manufacturer’s instructions. FPKM of each gene was calculated and the read counts of each gene were obtained by HTSeq-count [31, 32]. Differential expression analysis was performed using the DESeq2 [33]. *P* value < 0.05 and foldchange > 2 or foldchange < 0.5 was set as the threshold for significantly differential expression gene (DEGs). Based on the hypergeometric distribution, GO and KEGG pathway enrichment analysis of DEGs were performed to screen the significant enriched term using R (v 3.2.0), respectively. The original RNA-seq data are available in Additional file 6.

### Histological analysis

At the end of the infection experiments, mice were euthanized to collect their tissues. Tissues were immediately fixed with 4% paraformaldehyde (w/v) and then embedded in paraffin. The embedded tissues were sectioned 5 µm thick. These sections were then stained with hematoxylin and eosin (H&E) according to the manufacturer's (Beyotime Biotechnology Co., Ltd., China) instructions. Finally, these sections were scanned with NanoZoomer (Hamamatsu, Japan) and analyzed with Hamamatsu NDP view2 software.

### Immunohistochemistry

This study used ORIGENE kit to performed all immunohistochemistry experiments according to the manufacturer’s instructions (Beijing, China). In brief, slides were dewaxed and then incubated with specific antibodies according to recommended dilution at 4 ℃ for 12–16 h. Then, slides were washed with PBS and incubated with HRP-conjugated secondary antibodies for 40–60 min at room temperature. Finally, slides were visualized using DAB staining and then counterstained with hematoxylin. The microscopic image was captured by an optical microscope (OLYMPUS, Japan). The image analysis was conducted in 3 random sections from 1 representative mice, and total of 3 representative mice were analyzed in an animal experiment.

### In situ TUNEL Staining

Tunel Staining was performed according to the manufacturer’s instructions of One-step TUNEL In Situ Apoptosis Kit (Elabscience, China). Briefly, dewaxed slides were incubated with FITC-conjugated nucleotide and terminal deoxynucleotide transferase (TdT), followed by incubation with DAPI. The microscopic image was captured by confocal microscopy. The apoptotic cells were stained with FITC.

### Enzyme-linked immunosorbent assay (ELISA)

Briefly, serum samples were used for ELISAs according to the manufacturer’s protocol for the Mouse ELISA Kit with Plates (88–7064-86 for IL-6, 88–7013-22 for IL-1β, and 88–7013-22 for TNF-α, Invitrogen, USA).

### Generation of lentivirus

Lentivirus was generated by co-transfection with package plasmids pMDG.2 and psPAX2 and shRNA plasmid pLKO.1 on HEK293FT cells using Lipo3000 transfection agent (Thermo Fisher, USA). The sequence information of shRNA was provided in Additional file 2.

### Bacterial colonization

Tissues were homogenized aseptically in 1 mL of saline and diluted into triplicate on Martin’s broth agar, followed by cultivation at 37 ℃ for 16–20 h. The number of bacterial colonization was recorded through counting the average of CFU on these agar plates.

### Statistical analysis

Data were analyzed by GraphPad Prism software (Prism 6.0) and PASW Statistical 18.0 software (SPSS). Kaplan–Meier analysis (Prism 6.0) was used to analyzed mouse survival curves. Unpaired Student’s *t*-test (Prism 6.0) was used to study the difference between the two groups. One-way ANOVA was used to evaluate the difference between ≥ 3 groups, followed by Tukey multiple comparison testing. Groups of individual data are expressed as the means ± standard deviation (SD). Statistical significance was represented as followed. *: *p* < 0.05, **: *p* < 0.01, ***: *p* < 0.001.

## Results

### Apoptosis of pulmonary epithelial cells induced by *Pasteurella multocida* is essential for pathogenesis

**Figure 1:**
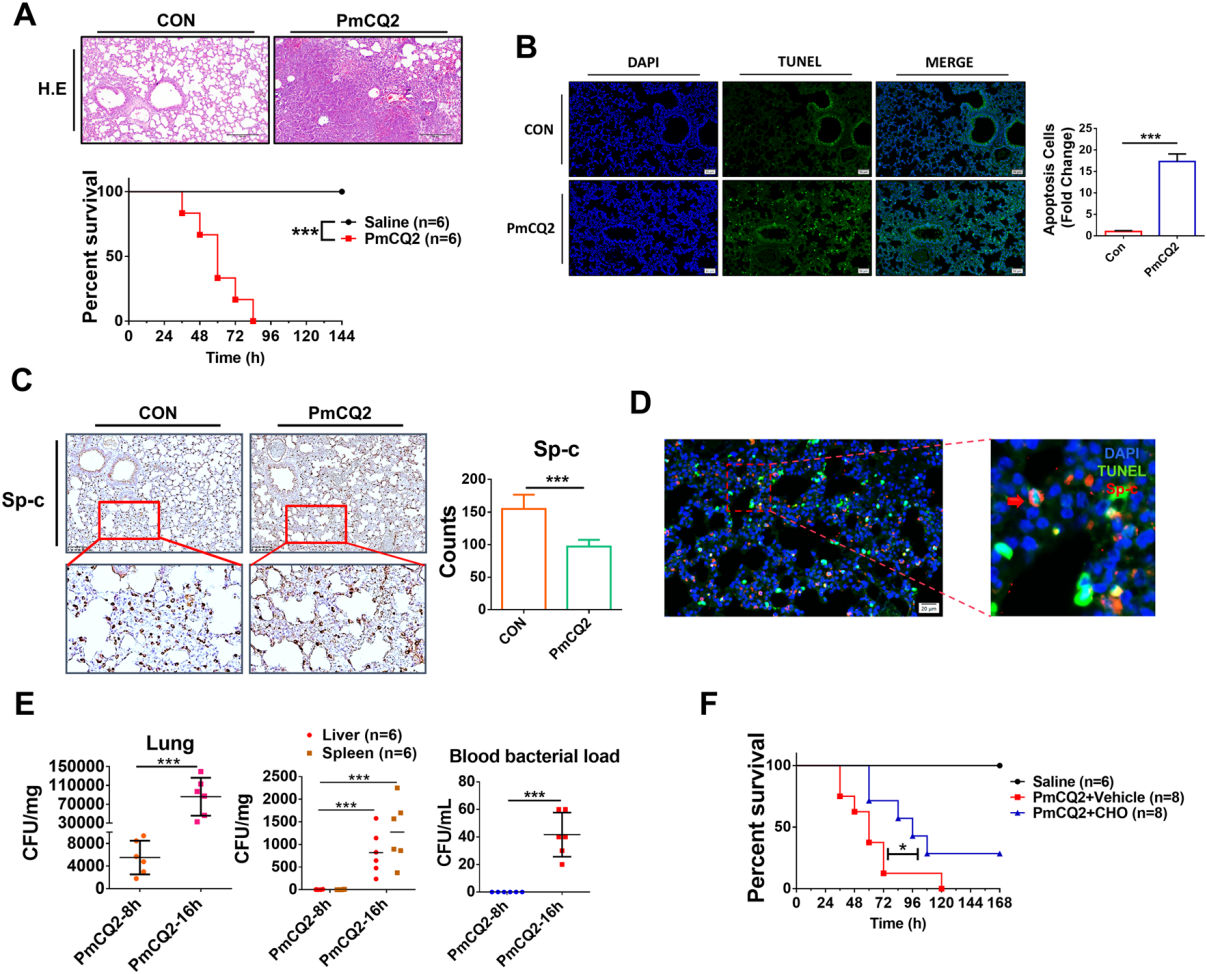
***Pasteurella multocida*-induced pulmonary epithelial apoptosis is critical for its pathogenesis.** **A** Mice were infected intranasally with 10^4^ CFU PmCQ2 for 32 h. Scale bar = 100 µm. Bottom is the survival curve of mice infected intranasally with 10^4^ CFU PmCQ2. **B** TUNEL staining of wild-type mouse lungs or 10^4^ CFU PmCQ2 mouse lungs at 16 h. The right panel shows the number of TUNEL-positive cells in 3 random sections. **C** Immunohistochemical staining of Sp-c in the lungs of wild-type mice and Sp-c in the lungs of mice intranasally infected with 10^4^ CFU PmCQ2 at 16 h post-infection (hpi). The graph on the right shows the number of Sp-c-positive cells in 3 random sections. **D** Representative images of confocal analysis of Sp-c staining and TUNEL staining in lungs of mice infected in situ with 10^4^ CFU PmCQ2 or saline at 16 hpi. Zoom bar = 50 μm. Red arrows indicate apoptotic lung epithelium. **E** Bacterial load in the lungs, liver, kidneys and bloodstream of mice infected with 10^4^ CFU PmCQ2 at 16 hpi. **F** For Ac-DEVD-CHO (CHO) treatment, mice were injected intranasally with 2 mg/kg CHO 30 min before infection. Survival curves of PmCQ2-infected mice with or without CHO treatment. Each group consists of 8 mice. Each point represents one individual. *p < 0.05, ***p < 0.001.

As shown in Figure 1A and Additional file 1A, consistent with clinical cases, PmCQ2 infection resulted in lethal lung injury in both mouse and rabbit models, with destruction of alveolar tissue, including epithelial cell detachment and damage. It was also found that a large number of apoptotic cells and inflammatory cells were present in the infected lungs (Figure 1B; Additional files 1B and C), which is consistent with previous reports [16, 17]. Notably, we found that the number of Sp-c-positive cells belonging to the lung epithelium was significantly reduced after PmCQ2 infection (Figure 1C), and then we confirmed the presence of many apoptotic lung epithelia in PmCQ2-infected lungs by Sp-c and TUNEL co-staining (Figure 1D). In addition, we found that PmCQ2 not only infected the lungs, but also extrapulmonary organs such as the liver, kidney and spleen at a later stage, and this extrapulmonary infection was accompanied by bacteremia (Figure 1E). These results suggest that Pm disrupts the pulmonary epithelial barrier and induces bacteremia and extrapulmonary infections. Importantly, we demonstrated that intranasal injection of the apoptosis inhibitor Ac-DEVD-CHO significantly attenuated PmCQ2 infection in a mouse model (Figure 1F), suggesting that Pm-induced excessive apoptosis in the lungs is one of the pathogenic mechanisms. Taken together, these results suggest that Pm-induced apoptosis of pulmonary epithelial cells plays a key role in its pathogenesis.

### *Pasteurella multocida* activates the Hippo-Yap pathway

**Figure 2:**
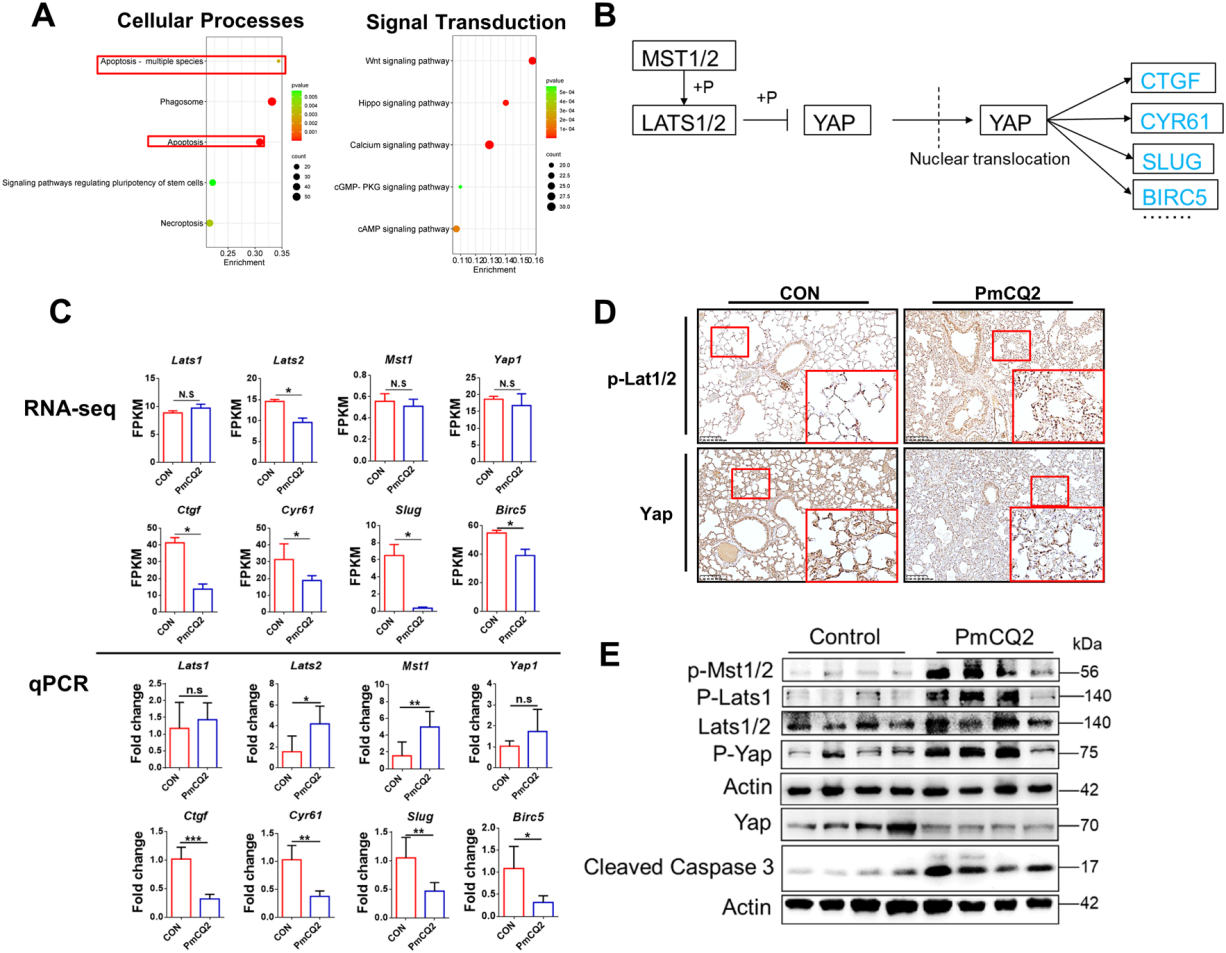
***Pasteurella multocida*** **activates the Hippo-Yap pathway.** **A** On the left, the first 5 RNA-seq-enriched pathways and the first 5 down-regulated signaling pathways of cellular processes in mouse lungs after infection with 10^4^ CFU PmCQ2 at 24 hpi. **B** Hippo-Yap pathway analysis in RNA-seq. Blue boxes indicate significant down-regulation. **C** Analysis of the Hippo pathway core components at the mRNA level. The upper part was analyzed by RNA-seq. The lower part was validated by q-PCR. q-PCR was performed on 5 individuals per group at 16 hpi. **D** Immunohistochemical staining of p-Lats1 and Yap in the lungs of wild-type and mice infected with 10^4^ CFU of PmCQ2 for 16 h. Scale bar = 100 µm. **E** Western blotting to analyze the dynamics of the Hippo pathway in the lungs of wild-type and mice infected with 10^4^ CFU PmCQ2 for 16 h. *p < 0.05, **p < 0.01, ***p < 0.001, N.S: no significant.

To further investigate the intrinsic mechanism of Pm-activated apoptosis in pulmonary epithelial cells, we performed RNA sequence analysis in mouse lungs. The results revealed that the apoptotic pathway was among the top five enriched cellular processes, suggesting that apoptosis is critical for Pm infection (Figure 2A). Furthermore, consistent with our previous work [13], we found that the Hippo pathway, which is closely associated with activation of apoptosis, was one of the five pathways with the most down-regulated expression in RNA-seq (Figure 2A), suggesting that the Hippo pathway may be involved in Pm-induced apoptosis in pulmonary epithelial cells. Notably, the expression of a series of downstream effectors of the Hippo pathway, such as *Cyr61* and *Birc5*, were significantly down-regulated in the RNA-seq data, whereas the expression of key components of the Hippo pathway, such as Mst1, Lats1/2, and the transcription factor Yap, remained unchanged (Figure 2B; Additional files 2A and B), suggesting that Pm could activate the Hippo pathway. These RNA-seq results were further confirmed by q-PCR (Figure 2C). In addition, immunohistochemical examination showed that PmCQ2-induced accumulation of phosphorylated Lats1 was associated with reduced Yap expression in the lungs, suggesting that Hippo-mediated Yap degradation was activated (Figure 2D). Similarly, Western blotting results revealed significant accumulation of phosphorylated Mst1/2, phosphorylated Lats1, and phosphorylated Yap in the lungs of Pm-infected mice (Figure 2E), suggesting activation of the Hippo pathway by Pm. In addition, Yap downstream effector Slug, which is involved in anti-apoptosis [27], was down-regulated during Pm infection (Additional file 2C), suggesting that Pm infection inhibited the transcriptional activity of Yap. Thus, the above data suggest that Pm does activate the Hippo-Yap pathway.

### *Pasteurella multocida* activation of the Hippo-Yap pathway causes pulmonary epithelial apoptosis

**Figure 3:**
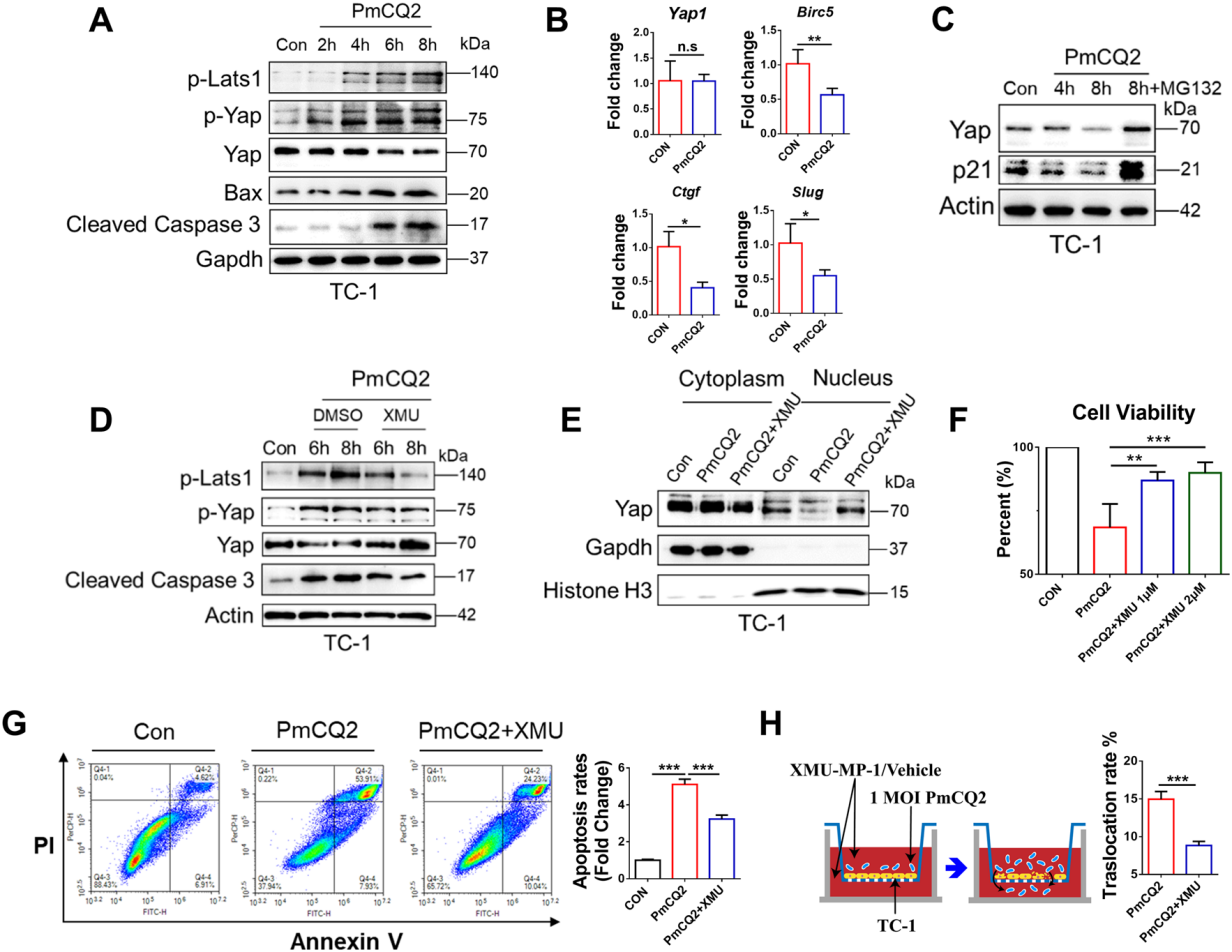
***Pasteurella multocida *activation of the Hippo-Yap pathway causes pulmonary epithelial apoptosis.** **A** Western blot analysis of the dynamics of the Hippo-Yap pathway in mouse lung epithelial cells after TC-1 infection with 1 MOI PmCQ2. **B** Analysis of Yap expression at the mRNA level in mouse lung epithelial cells 8 h after TC-1 infection with 1 MOI PmCQ2. **C** Western blot analysis of mouse lung epithelial cells TC-1 infected with 1 MOI PmCQ2 with or without 10 µM proteasome inhibitor MG-132. **D** Western blot analysis of the dynamics of the Hippo-Yap pathway in mouse lung epithelial cells TC-1 infected with 1 MOI PmCQ2 plus vector or 1 µM XMU-MP-1 for 6 or 8 h. **E** Western blot analysis of Yap subcellular localization in TC-1 infected with 1 MOI PmCQ2 with or without 1 µM XMU-MP-1 for 8 h. **F** Cell viability analysis of TC-1 cells infected with 1 MOI PmCQ2 plus vector or different concentrations of XMU-MP-1 for 10 h. Cell viability was estimated by LDH levels in the cell. **G** Analysis of apoptosis rate of TC-1 cells infected with 1 MOI PmCQ2 plus vector or different concentrations of XMU-MP-1 for 10 h. The right panel shows the quantification of apoptosis-positive cells. **H** A scheme presents the Transwell assay protocol. At the right are quantifications of translocated bacteria count of TC-1 cells treated with 1 µM XMU-MP-1 or Vehicle for 8 h. Each group contained 3 replicates. **p < 0.01, ***p < 0.001, N.S: no significant.

It is well known that the Hippo pathway controls cell growth and survival. The state of the Hippo pathway in lung epithelial cells is also closely related to tissue repair, regeneration and apoptosis [20]. Given that Pm activated the Hippo-Yap pathway in vivo, we next determined whether Pm could directly activate the Hippo pathway in lung epithelial cells in vitro. Figure 3A shows that the activation of the Hippo pathway in the lung epithelial cell line TC-1 by Pm resulted in the accumulation of phosphorylated Lats1 and Yap, accompanied by a decrease in Yap expression. Furthermore, the activation of the Hippo pathway also led to an upregulation of the apoptosis-executing protein Cleaved Caspase 3. In addition, we examined the mRNA expression levels of Yap and its downstream genes. Transcriptional levels of Yap-inducible genes such as *Cyr61* and *Slug* were downregulated after PmCQ2 infection, which is consistent with the in vivo results (Figure 3B). Notably, PmCQ2 infection did not reduce *Yap* mRNA level, suggesting that Yap downregulation is a post-transcriptional regulation process. Given that proteasomal clearance of Yap is the final step of the Hippo pathway, we used the proteasome inhibitor MG-132 to block proteasomal activity. As expected, inhibition of the proteasome activity (evidenced by the accumulation of p21 [34]) rescued Yap expression under Pm infection (Figure 3C), suggesting that the degradation of Yap occured through the proteasome pathway. These results suggest that Pm directly activates the Hippo pathway in pulmonary epithelial cells in vitro. We further validated the role of the Hippo pathway in pulmonary epithelial cells during Pm infection using XMU-MP-1 (XMU), a Hippo pathway inhibitor that reduces the activity of Mst1/2. Western blotting showed that inhibition of Mst1/2 resulted in the accumulation of Yap and a decrease in Cleaved Caspase 3 (Figure 3D; Additional file 3). Considering that the cytoplasmic localization of Yap is a hallmark of the Hippo pathway activation, we also found that PmCQ2 significantly reduced the nucleus localization of Yap, while XMU-MP-1 aborted this effect (Figure 3E). Furthermore, as shown in Figure 3F, inhibition of the Hippo pathway significantly increased the survival of TC-1 cells, suggesting that Pm-induced activation of the Hippo-Yap pathway may be involved in promoting host cell death. To this end, we further investigated the apoptosis of TC-1 cells during Pm infection with or without XMU-MP-1 using flow cytometry. As a result, PmCQ2-induced apoptosis was significantly reduced after XMU-MP-1 treatment (Figure 3G), suggesting that the Hippo-Yap pathway contributes to Pm-induced apoptosis in pulmonary epithelial cells. Importantly, we further confirmed that inhibition of the Hippo-Yap pathway attenuated Pm-induced lung epithelial barrier loss by transwell model (Figure 3H). Thus, the above results suggest that the Hippo-Yap pathway contributes to Pm-induced apoptosis in pulmonary epithelial cells.

### Pharmaceutical inhibition of the Hippo-Yap pathway attenuates *Pasteurella multocida* infection

**Figure 4:**
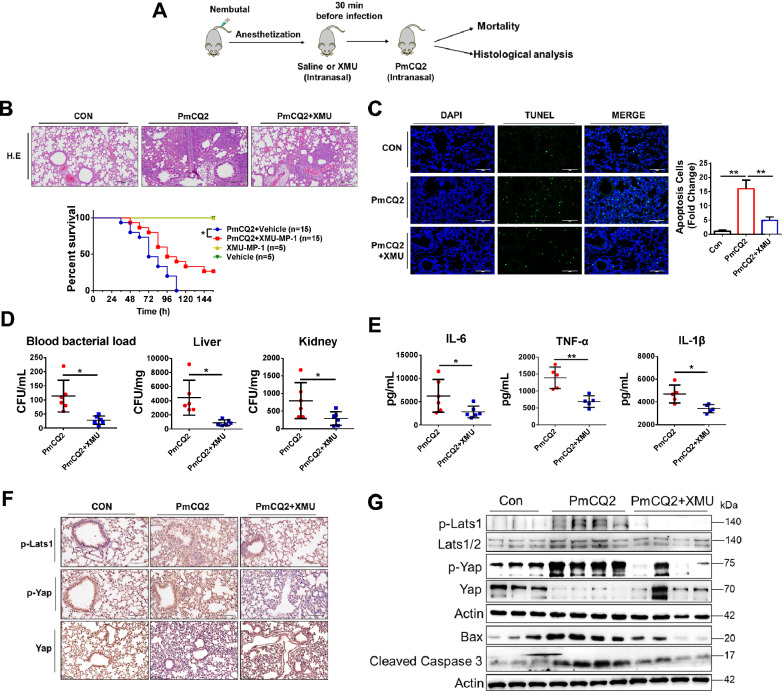
**Pharmaceutical inhibition of the Hippo-Yap pathway attenuates * Pasteurella multocida*** **infection.A** Scheme presenting the XMU-MP-1 (XMU) treatment protocol. **B** H&E staining of the lungs of control, 10^4^ CFU PmCQ2-infected and 10^4^ CFU PmCQ2 plus 3 mg/kg XUM-MP-1 infected mice. Mice were infected for 32 h. Scale bar = 100 µm. The graph below shows the survival rates of control, 10^4^ CFU PmCQ2-infected, and 10^4^ CFU PmCQ2-plus 3 mg/kg XUM-MP-1-infected mice. **C** TUNEL staining of lungs from control, 10^4^ CFU PmCQ2-infected and 10^4^ CFU PmCQ2 plus 3 mg/kg XUM-MP-1-infected mice. Scale bar = 100 µm. The right panel shows the quantification of TUNEL-positive cells in 3 random sections. **D** Bacterial load in mice infected with 10^4^ CFU PmCQ2 and mice infected with 10^4^ CFU PmCQ2 plus 3 mg/kg XUM-MP-1. Mice were infected for 16 h. **E** Quantification of IL-6, TNF-α, and IL-1β in the serum of PmCQ2-infected mice and PmCQ2-infected mice with XMU-MP-1 treatment at 16 hpi by ELISA. **F** Immunohistochemistry staining of p-Lats1, p-Yap and Yap in mice lungs infected with 10^4^ CFU PmCQ2 group and infected with 10^4^ CFU PmCQ2 plus XUM-MP-1 group. Scale bar = 200 µm. **G** Western blot analysis of the dynamics of Hippo pathway infection in 10^4^ CFU PmCQ2 group and 10^4^ CFU PmCQ2 plus XUM-MP-1 group mice at 16 hpi. *p < 0.05, **p < 0.01.

To further investigate the role of the Hippo-Yap pathway in vivo, we inhibited Mst1/2 activity by intranasal injection of 3 mg/kg XMU-MP-1 30 min before PmCQ2 challenge (Figure 4A). As a result, PmCQ2-induced lung lesions were significantly reduced after XMU-MP-1 treatment, which further coincided with the increase in survival (Figure 4B). Apoptotic cells in situ in infected lungs were significantly reduced after XMU-MP-1 treatment (Figure 4C; Additional file 4A). Consistent with the reduction in apoptotic cells, there was a corresponding significant reduction in the amount of bacteria in the blood, liver, and kidneys, as well as indices of liver and kidney damage (aspartate aminotransferase (AST), alanine aminotransferase (ALT), creatinine (CREA), and urea nitrogen (BUN) (Figure 4D; Additional file 4B). XMU-MP-1 treatment also significantly downregulated the systemic inflammatory factors IL-6, TNF-α, and IL-1β (Figure 4E). These results indicate that XMU-MP-1 treatment had a protective effect against Pm infection. In addition, immunohistochemical examination showed that XMU-MP-1 treatment indeed inhibited the Hippo-Yap pathway, which was reflected by the reduction of p-Lats1 and p-Yap and the upregulation of Yap (Figure 4F). Similarly, Western blotting results confirmed that XMU-MP-1 inhibited the Hippo-Yap pathway, rescued Yap expression, and reduced the apoptosis execution factor Cleaved Caspase 3 in lungs (Figure 4G). These results suggest that Pm-mediated activation of the Hippo-Yap pathway is one of the pathogenetic mechanisms leading to pasteurellosis and that pharmacological inhibition of the Hippo pathway could be a potential strategy for the treatment of pasteurellosis.

### *Pasteurella multocida* activates the Hippo-Yap pathway by inducing Rassf1 expression

**Figure 5:**
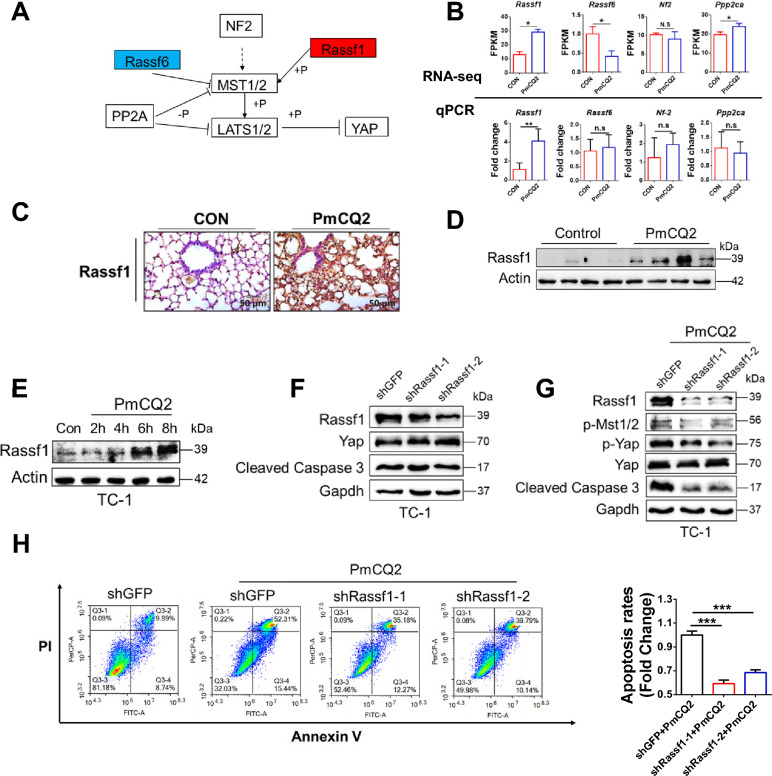
***Pasteurella multocida *activates the Hippo-Yap pathway by inducing Rassf1 expression.** **A** Analysis of Hippo-Yap pathway regulators based on RNA-seq data. Blue boxes indicate significant decreases and red boxes indicate significant increases. **B** Analysis of Hippo pathway regulators at the mRNA level. The upper part was analyzed by RNA-seq. The lower part was validated by q-PCR results. Each q-PCR group consists of 5 individuals at 16 hpi. **C** Immunohistochemical staining of Rassf1 in mouse lungs. Mice were infected with 10^4^ CFU PmCQ2 for 16 h. Scale bar = 100 µm. **D** Western blot analysis of Rassf1 in mouse lungs. Mice were infected with 10^4^ CFU PmCQ2 for 16 h. **E** Western blot analysis of Rassf1 in TC-1 cells infected with 1 MOI PmCQ2. **F** Western blot analysis of TC-1 cells based on shRNA knockdown of Rassf1. **G** Western blot analysis of TC-1 cells infected with 1 MOI PmCQ2 based on shRNA knockdown of Rassf1. **H** Analysis of apoptosis rates of TC-1 cells infected with 1 MOI PmCQ2 plus shGFP or shRassf1 at 10 hpi. On the right is quantitation of apoptosis positive cells. ***p < 0.001.

**Figure 6:**
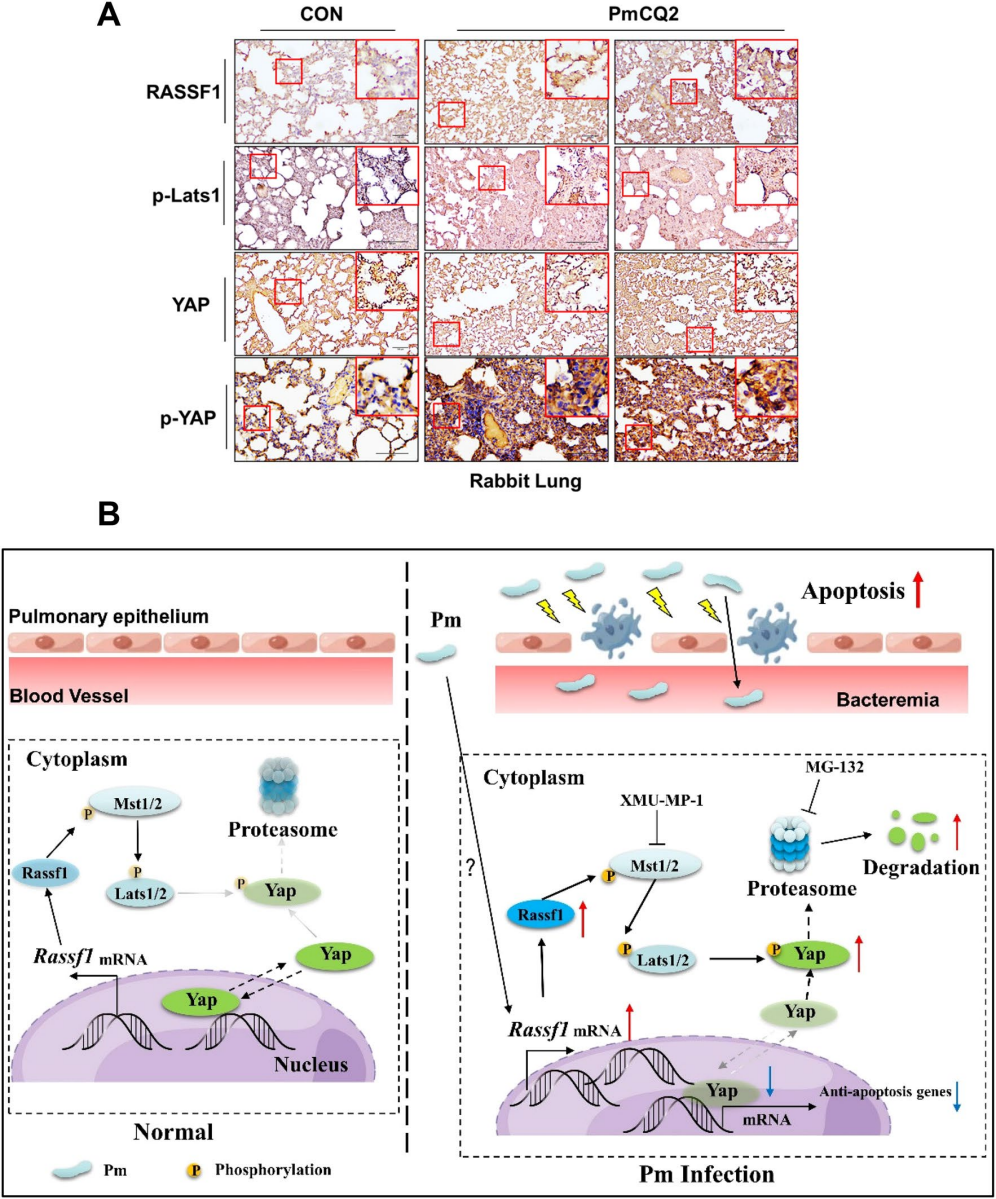
***Pasteurella multocida *activates the Rassf1-Hippo-Yap pathway in rabbit models.** **A** Immunohistochemical staining for Rassf1, p-Lats1, p-Yap, and Yap in healthy and infected rabbit lungs with 10^9^ CFU PmCQ2 for 36 h. Scale bar = 200 µm. For p-Yap, Scale bar = 100 µm. **B** *Pasteurella multocida* manipulates the Hippo-Yap pathway in lung epithelial cells through transcriptional regulation of Rassf1, leading to degradation of Yap via the proteasome and inducing excessive apoptosis in pulmonary epithelial cells. In contrast, pharmacological inhibition of the Hippo-Yap pathway by XMU-MP-1 rescued the transcriptional function of Yap, thereby attenuating Pm-induced lung injury and reducing animal mortality.

Many proteins and pathways are able to activate the Hippo pathway, including mechanical signal, G protein signal and some phosphatases [23]. To find a potential mechanism for Pm activation of the Hippo pathway, we again reviewed RNA-seq data and found that two phosphatases, Rassf1 and Rassf6, may be involved. Rassf1 phosphorylates MST1/2 and thus activates the Hippo pathway [35], while Rassf6 restricts the activity of MST1/2, leading to Hippo pathway inhibition [36]. In our RNA-seq data, Rassf1 is significantly enriched in RNA-seq and Rassf6 is significantly down-regulated (Figure 5A). However, q-PCR results only revealed that Rassf1 was significantly up-regulated during PmCQ2 infection, whereas Rassf6 was not significantly changed compared with the wild type (Figure 5B). In addition, both Western blotting and IHC results showed that Rassf1 accumulated in PmCQ2-infected lungs and TC-1 (Figures 5C–E). Therefore, Pm-induced Rassf1 upregulation might be involved in the activation of the Hippo-Yap pathway. To this end, we knocked down Rassf1 in vitro and detected the Hippo-Yap pathway. As shown in Figures 5F and G, knockdown of Rassf1 during PmCQ2 infection resulted in downregulation of p-Mst1 and p-Yap and decreased cleaved Caspase 3. Furthermore, flow cytometry results showed that knockdown of Rassf1 also protected TC-1 from PmCQ2-induced apoptosis (Figure 5H). Thus, these results suggest that Pm activates the Hippo-Yap pathway by up-regulating the expression of Rassf1. As Pm is usually isolated from domestic animals, including rabbits, cows, and chickens, we further explored whether clinical samples from rabbits showed a similar trend to the mouse results. As shown in Figure 6A, the lungs of PmCQ2-infected rabbits displayed accumulation of Rassf1, p-Lats1, and p-Yap, which was consistent with the results in mice, reaffirming that Pm can activate the Rassf1-Hippo-Yap pathway to induce apoptosis of lung epithelial cells in the clinical samples. The hypothetical graph is shown in Figure 6B.

## Discussion

*Pasteurella multocida* is an important respiratory pathogen with a wide range of host types, including animals and humans. Moreover, the number of Pm infections in humans has increased significantly over the past two decades, making Pm a potential public health risk factor [11]. However, the mechanisms by which Pm induces pneumonia and respiratory syndromes are largely unknown. Apoptosis is a mode of programmed cell death, and its disruption is closely associated with the development of many infectious diseases [37]. Previous pathologic studies have found that loss of structural integrity of epithelial cells due to cell death and large numbers of apoptotic cells can be observed in Pm infected lungs [12, 38–40]. However, how Pm leads to apoptosis in pulmonary epithelial cells remains largely unknown.

Due to the lack of effective vaccines and potent drugs, antibiotics are still the most commonly used drugs for the clinical treatment of Pm infections. Therefore, there is an urgent need for researchers to further discover the underlying pathogenesis and potential drug targets. It has been shown that Pm infection leads to massive apoptosis in tissues, and our previous studies also found that the apoptotic pathway was significantly enriched in Pm-infected lungs, implying that apoptosis is closely related to Pm infection [9, 13, 16, 17]. In addition, due to excessive cell death, a large number of immune cells are continuously recruited to the lesion site, leading to an exaggerated inflammatory response. The Hippo pathway is a highly conserved evolutionary signaling pathway that plays an important role in tissue development, repair, regeneration, cell proliferation, and survival [18, 19]. Previous studies have found that the Hippo pathway plays an important role during infection [21, 41, 42]. For example, during COVID-19 infection, the Hippo pathway is activated and promotes host antiviral resistance by limiting viral replication [21]. Zika virus alters DNA methylation and initiates the Hippo pathway, which regulates host antiviral immunity and apoptosis [41, 42]. In *Streptococcus pneumoniae* infection and LPS-induced lung injury, the Hippo pathway has been associated with the alleviation of lung inflammation and the repair and regeneration of alveolar epithelium [20, 30]. The Hippo pathway also exerts an antimicrobial effect by inducing more ROS to promote the bactericidal activity of macrophages [43]. Yap is a key downstream effector of the Hippo pathway and is closely associated with cell proliferation and anti-apoptosis [44]. It is well known that intestinal epithelial cell death, including apoptosis, leads to intestinal barrier disruption and overproduction of inflammatory factors [45]. Similarly, Pm-induced apoptosis of a large number of lung epithelial cells can lead to increased lung permeability and consequently extrapulmonary infections. Notably, Pm bacteremia and Pm-induced extrapulmonary injury are becoming more common in clinical conditions [46–48], suggesting that Pm-induced pulmonary permeability via inducing excessive epithelial apoptosis could be an important part of its pathogenesis. Indeed, our recent studies also indicate that a complete model of Pm infection consists of at least two phases, pulmonary infection phase and extrapulmonary infection phase, and that extrapulmonary infection phase contribute to the Pm-induced cytokine storm and is critical for host death (data not shown). Therefore, understanding whether the Hippo pathway is involved in Pm-mediated apoptosis in pulmonary epithelial cells is essential for pasteurellosis.

In this study, using unbiased RNA-seq, we found that a series of Hippo pathway downstream effectors were down-regulated in the lungs of Pm-infected mice, suggesting that Pm activates the Hippo-Yap pathway, which was further confirmed by experiments in cell culture models and animal models. We found that activation of the Hippo pathway (inactivation of Yap) may disrupt homeostasis in the lung, leading to apoptosis of lung epithelial cells and amplification of the systemic inflammatory response. Indeed, blocking lung endothelial or epithelial YAP activity can exacerbate lung inflammation and impede its resolution [20, 49]. To further characterize the role of the Hippo pathway in Pm infection, we performed in vitro and in vivo assays using the Hippo pathway-selective inhibitor XMU-MP-1 in this study. The results revealed that inhibition of the Hippo-Yap pathway had an anti-infection effect, and XMU-MP-1 significantly increased cell viability and decreased apoptosis in the mouse lung epithelial cell line TC-1 in vitro, as well as significantly reduced lung lesions, apoptosis, inflammatory factor levels and mortality in vivo. Thus, the Hippo-Yap pathway could be a potential target for controlling Pm infection. In addition, we identified a potential mechanism by which Pm activates the Hippo-Yap pathway. Considering that p-Yap does not increase at first, but increases significantly after 4–6 h of Pm infection in vitro, we suggested that the Hippo-Yap pathway may be transcriptionally regulated. Therefore, we reviewed RNA-seq data and found that the tumor suppressor gene Rassf1 was significantly up-regulated, and that Rassf1 enhanced Mst1/2 activity by phosphorylation [35]. Accordingly, knockdown of Rassf1 rescued Yap activity and inhibited Pm-induced apoptosis, suggesting that upregulated Rassf1 is involved in Pm-induced activation of the Hippo-Yap pathway. However, the present study did not reveal the exact mechanism of Pm-induced Rassf1 expression. A previous study showed that Zika virus can regulate Rassf1 expression by altering the methylation level of the promoter [42]. Therefore, our next work needs to investigate the exact mechanism by which Pm upregulates Rassf1. Finally, we found that the expression of Rassf1, p-Lats1 and p-Yap was also increased in rabbit samples, implying that targeting the Hippo pathway has potential clinical value.

In conclusion, our current work demonstrated that Pm infection induces activation of the Hippo-Yap pathway through upregulation of Rassf1 expression. Activation of the Rassf1-Hippo-Yap pathway led to Pm-induced apoptosis in lung epithelial cells. In contrast, pharmacological inhibition of the Hippo pathway rescued cell viability and reduced apoptosis, lung injury, and animal mortality. Thus, activation of the Hippo-Yap pathway contributes to the pathogenesis of Pm, and targeting the Hippo-Yap pathway is a potential approach to controlling pasteurellosis.

### Supplementary Information

**Additional file 1. PmCQ2-induced lung injury and apoptosis in rabbits.** (A) H&E staining of infected rabbit lungs. Scale bar = 200 µm. (B) TUNEL staining of infected rabbit lungs. Scale bar = 100 µm. (C) GR-1 staining of infected mouse lungs. Scale bar = 100 µm.

**Additional file 2. RNA-seq analysis of the Hippo-Yap pathway in the lungs of infected mice.** (A) Kegg (Kyoto Encyclopedia of Genes and Genomes) pathway enrichment of the Hippo-Yap pathway in the lungs of infected mice. Green boxes indicate significant down-regulation and red boxes indicate significant up-regulation. (B) Expression of downstream effectors of Yap in the Kegg pathway. (C) Western blot analysis of Yap downstream effector Slug in the lungs of PmCQ2-infected mice.

**Additional file 3. Western blot analysis of Hippo pathway proteins in LLC cells.**

**Additional file 4. Analysis of XMU treated mice. (A) Sp-c and TUNEL co-staining in XMU treated mouse lungs.** (B) Inhibition of the Hippo pathway alleviates liver and kidney injury indices in PmCQ2-infected mice.

**Additional file 5. Information of reagent, antibody, plasmid and primer used in this study.**

**Additional file 6. The original RNA-seq data.**

## Data Availability

The data used to support the findings of this study are available from the corresponding author upon reasonable request.
