# Supplementary figures and images for: Pasteurella multocida activates Rassf1-Hippo-Yap pathway to induce pulmonary epithelial apoptosis
Source: Vet Res. 2024 Mar 16;55:31. doi: 10.1186/s13567-024-01285-y (PMC10943858; doi:10.1186/s13567-024-01285-y)

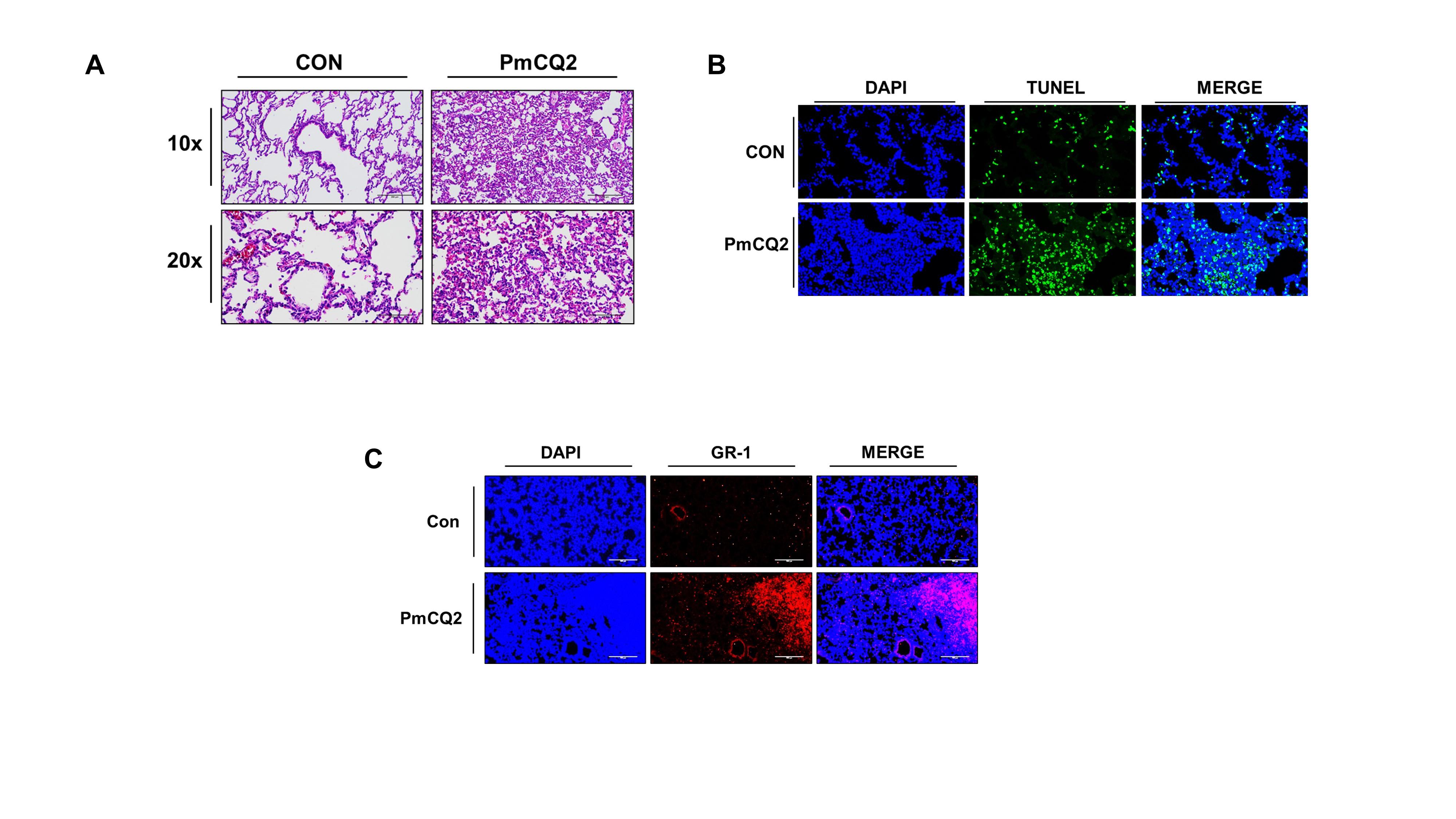

Supplement: Supplementary file 1 — Additional file 1. PmCQ2-induced lung injury and apoptosis in rabbits. (A) H&E staining of infected rabbit lungs. Scale bar = 200 µm. (B) TUNEL staining of infected rabbit lungs. Scale bar = 100 µm. (C) GR-1 staining of infected mouse lungs. Scale bar = 100 µm. [file 13567_2024_1285_MOESM1_ESM.jpg]

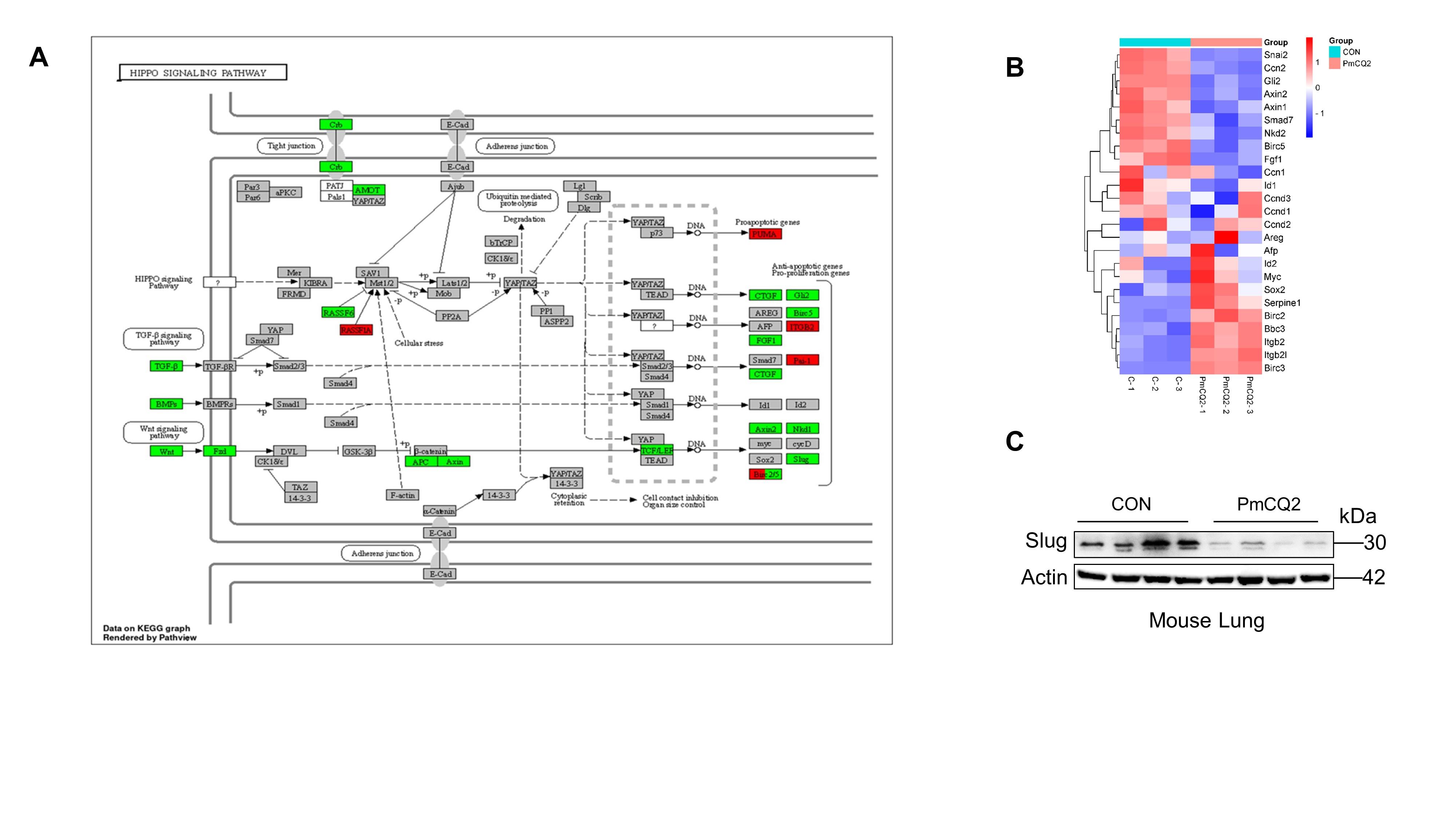

Supplement: Supplementary file 2 — Additional file 2. RNA-seq analysis of the Hippo-Yap pathway in the lungs of infected mice. (A) Kegg (Kyoto Encyclopedia of Genes and Genomes) pathway enrichment of the Hippo-Yap pathway in the lungs of infected mice. Green boxes indicate significant down-regulation and red boxes indicate significant up-regulation. (B) Expression of downstream effectors of Yap in the Kegg pathway. (C) Western blot analysis of Yap downstream effector Slug in the lungs of PmCQ2-infected mice. [file 13567_2024_1285_MOESM2_ESM.jpg]

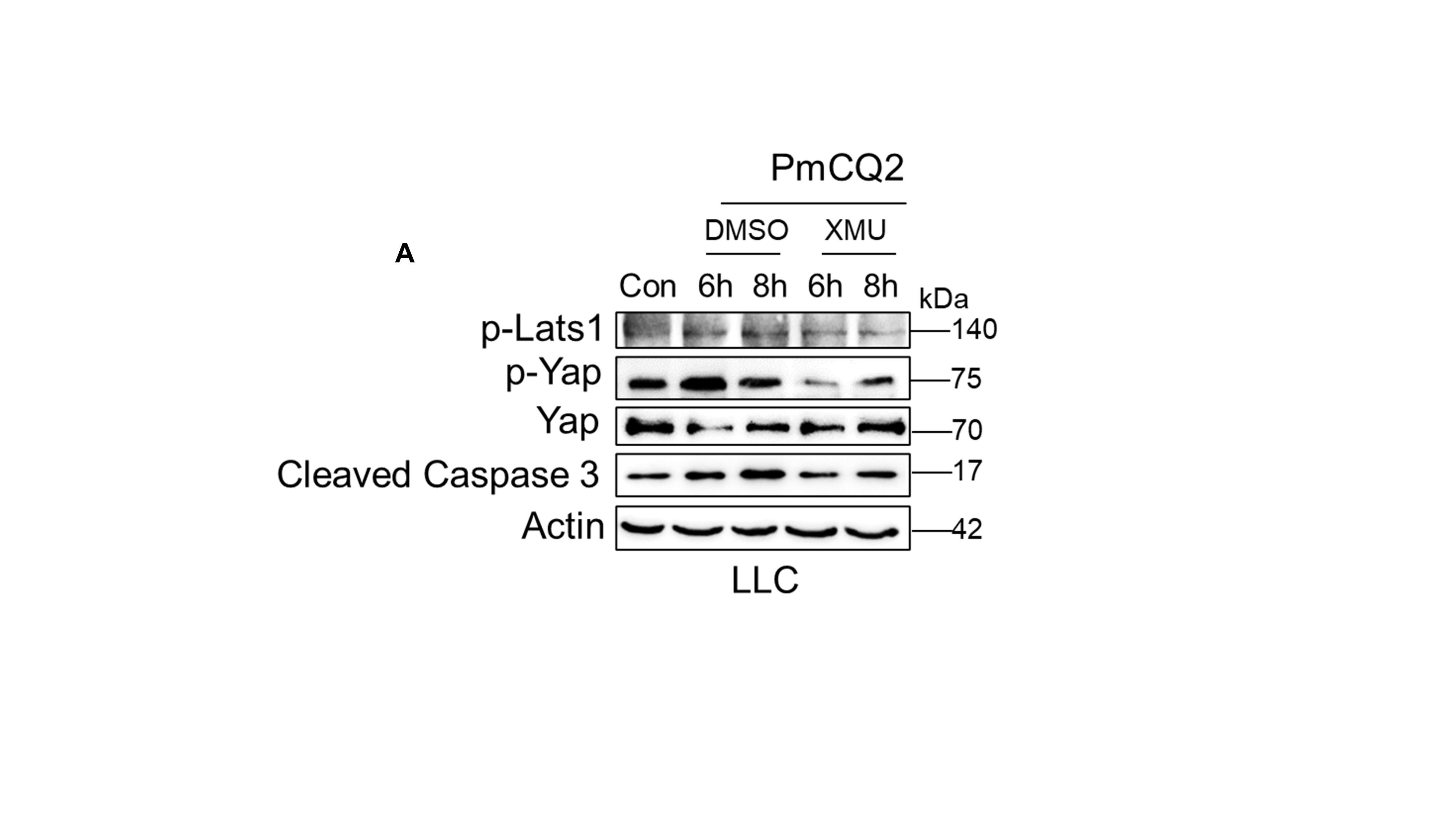

Supplement: Supplementary file 3 — Additional file 3. Western blot analysis of Hippo pathway proteins in LLC cells. [file 13567_2024_1285_MOESM3_ESM.jpg]

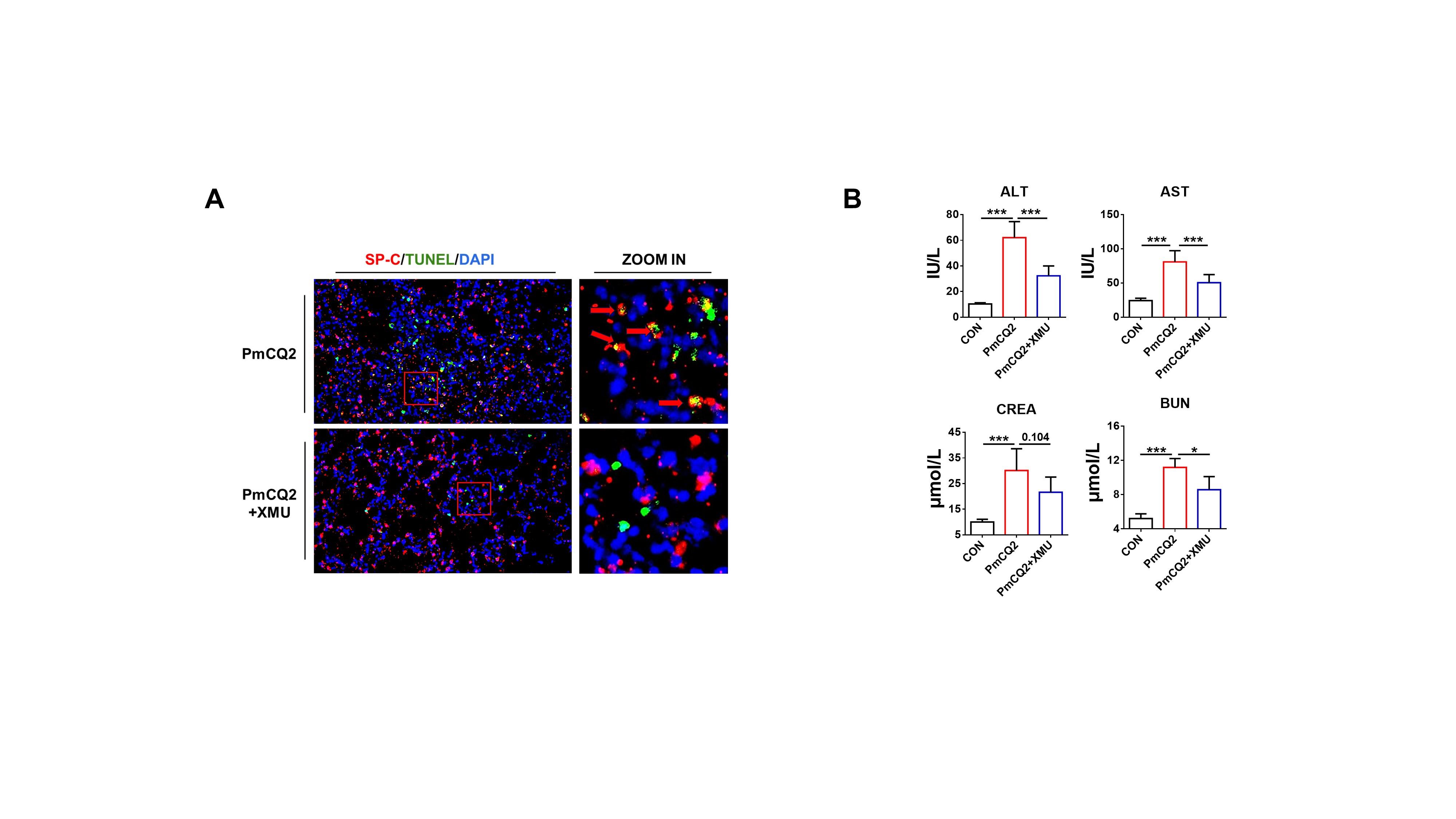

Supplement: Supplementary file 4 — Additional file 4. Analysis of XMU treated mice. (A) Sp-c and TUNEL co-staining in XMU treated mouse lungs. (B) Inhibition of the Hippo pathway alleviates liver and kidney injury indices in PmCQ2-infected mice. [file 13567_2024_1285_MOESM4_ESM.jpg]
