# Supplementary material for: Pasteurella multocida activates Rassf1-Hippo-Yap pathway to induce pulmonary epithelial apoptosis
Source: Vet Res. 2024 Mar 16;55:31. doi: 10.1186/s13567-024-01285-y (PMC10943858; doi:10.1186/s13567-024-01285-y)
Supplement: Supplementary file 5 — Additional file 5. Information of reagent, antibody, plasmid and primer used in this study. [file 13567_2024_1285_MOESM5_ESM.docx]

**Reagents and antibodies**

**Reagents:** Martin’s broth (Hopebio, China); H.E staining ((Biosharp, BL700A); DAB for IHC (Origene, ZLI-9018); DAB for IHC (Origene, ZLI-9018); MG-132 (MCE, HY-13259); XMU-MP-1 (CSNpharm, CSN18786); Paraformaldehyde (Sigma, 441244); Ac-DEVE-CHO (MCE, HY-P1001).

**Primary Antibodies:** YAP1 (Cell Signaling Technology, CST, 14074); p21 (Zenbio, 385235); Beta-ACTIN (Abclonal, AC026); GAPDH (Zenbio, 200306-7E4); S-pc (Proteintech, 10774-1-AP); p-MST1 (Thr183) /2 (Thr180) (Proteintech, 80093-1-RR), p-Lats1(Thr1079) (Immunoway, YP1383), Lats1/2 (Immunoway, YT2543); p-YAP (Ser397) (Immunoway, YP1554); Slug (Proteintech, 12129-AP-1); Cleaved-Caspase 3 p17 (Immunoway, YC0006); RASSF1 (Affinity, DF6335); BAX (Cell Signaling Technology, CST, 2772); Histone H3 (Cell Signaling Technology, CST, 4499);

**Secondary Antibodies:** Anti HRP-linked-rabbit IgG (Santa Cruz Biotechnology, sc2313); Anti HRP-linked-mouse IgG (Santa Cruz Biotechnology, sc2314); Anti HRP-linked-goat IgG (Santa Cruz Biotechnology, sc2033).

**Plasmids**

The pLKO.1, pMDG.2 and psPAX2 were stored in our laboratory. The shRNAs were cloned into pLKO.1. The sequence information of the primers used for shRNA were as following.

Rassf1-sh1: CCGGGCACTCTTTGAGCGAACTGAACTCGAGTTCAGTTCGCTCAAAGAGTGCTTTTTG

Rassf1-sh2: CCGGATCAAGGTTCAGCTGAAACTACTCGAGTAGTTTCAGCTGAACCTTGATTTTTTG

**Q-PCR primers**

Rassf6-F: CCTTCTTGGATCGTTGTCAATGA

Rassf6-R: CGTCTTCGGTCTGCCCATA

Nf2-F: CTAGTTCAAGAGATCACGCAACA

Nf2-R: GGCAGTAGACCTTTTCATCCAAA

Cyr61-F: CTGCGCTAAACAACTCAACGA

Cyr61-R: GCAGATCCCTTTCAGAGCGG

Snail2-F: TGGTCAAGAAACATTTCAACGCC

Snail2-R: GGTGAGGATCTCTGGTTTTGGTA

Ctgf-F: GGGCCTCTTCTGCGATTTC

Ctgf-R: ATCCAGGCAAGTGCATTGGTA

Lats1-F: AAAGCCAGAAGGGTACAGACA

Lats1-R: CCTCAGGGATTCTCGGATCTC

Lats2-F: GGACCCCAGGAATGAGCAG

Lats2-R: CCCTCGTAGTTTGCACCACC

Mst1-F: CGGGGTCCGTTTCAGACATAA

Mst1-R: GCGTTTTGCCATTGTATCTGTT

Ppp2ca-F: CGTTGTGGTAACCAAGCTGC

Ppp2ca-R: GCTGGGTCAAACTGCAAGAAAG

Birc5-F: GAGGCTGGCTTCATCCACTG

Birc5-R: CTTTTTGCTTGTTGTTGGTCTCC
